# Therapeutic lung lavages in children and adults

**DOI:** 10.1186/1465-9921-6-138

**Published:** 2005-11-22

**Authors:** Christian Paschen, Karl Reiter, Franz Stanzel, Helmut Teschler, Matthias Griese

**Affiliations:** 1 Dr. von Haunersches Kinderspital, University of Munich, Lindwurmstr. 4a, D-80337 Munich, Germany; 2 ASKLEPIOS Fachkliniken, Zentrum für Pneumologie und Thoraxchirurgie, Robert-Koch-Allee 2, D-82131 München-Gauting, Germany; 3 Ruhrlandklinik, Department Respiratory and Sleep Medicine, University of Essen, Tüschener Weg 40, Germany

**Keywords:** BAL, PAP, Protein

## Abstract

**Background:**

Pulmonary alveolar proteinosis (PAP) is a rare disease, characterized by excessive intra-alveolar accumulation of surfactant lipids and proteins. Therapeutic whole lung lavages are currently the principle therapeutic option in adults. Not much is known on the kinetics of the wash out process, especially in children.

**Methods:**

In 4 pediatric and 6 adult PAP patients 45 therapeutic half lung lavages were investigated retrospectively. Total protein, protein concentration and, in one child with a surfactant protein C mutation, aberrant pro-SP-C protein, were determined during wash out.

**Results:**

The removal of protein from the lungs followed an exponential decline and averaged for adult patients 2 – 20 g and <0.5 to 6 g for pediatric patients. The average protein concentration of consecutive portions was the same in all patient groups, however was elevated in pediatric patients when expressed per body weight. The amount of an aberrant pro-SP-C protein, which was present in one patient with a SP-C mutation, constantly decreased with ongoing lavage. Measuring the optical density of the lavage fluid obtained allowed to monitor the wash out process during the lavages at the bedside and to determine the termination of the lavage procedure at normal protein concentration.

**Conclusion:**

Following therapeutic half lung lavages by biochemical variables may help to estimate the degree of alveolar filling with proteinaceous material and to improve the efficiency of the wash out, especially in children.

## Introduction

Pulmonary alveolar proteinosis (PAP) is a rare respiratory disease characterized by the accumulation of surfactant-derived material in the lung of patients [1]. Currently PAP is categorized into acquired, congenital, and secondary PAP [2,3].

The acquired form of PAP is clinically characterized by cough, dyspnea and progression to respiratory failure. The presence of anti-GM-CSF auto-antibodies in serum and bronchoalveolar lavage (BAL) is of diagnostic value for this entity[3,4]. The congenital form of PAP is characterized by an acute onset immediately after birth with respiratory distress and rapid progression[5]. Pathogenetically mutations of the genes encoding surfactant protein B [6,7] and C[8,9], the GM-CSF receptor β subunit[10], or ABC-A3 [11] may lead to the accumulation of proteinaceous alveolar material. Secondary PAP is uncommon and includes cases with lysinuric protein intolerance, acute silicosis and other inhalational syndromes, immunodeficiency disorders, and malignancies and hematopoietic disorders[3].

Therapeutic bronchoalveolar lavages (BAL) are the principle option to reduce the abnormal accumulation of PAS positive proteinaceous material that fills the alveolar space of patients with pulmonary alveolar proteinosis (PAP)[3]. Little is known about the kinetics of the protein wash out during therapeutic whole lung lavages. The lavages of one adult patient were investigated by Onodera et al. and showed a rapid declining curve of protein and phospholipid in the successive lavage fractions[12]. Doyle et al. also showed the decrease of cholesterol, surfactant protein A (SP-A), surfactant protein B (SP-B) and phospholipids in aliquots[13]. Recently Perez and Rogers reported in adult patients that chest percussion therapy and positional changes during whole lung lavage enhanced alveolar clearance[14]. In children almost no data are available on the wash out kinetics.

The aim of the present study was to investigate the volume, the total amount, concentration and pattern of protein washed out of the lungs during such procedures in pediatric and adult patients with PAP and in one patient with cholesterol pneumonitis. The value of simple determination of the optical density (OD) to monitor the progress of the lavage procedure and to help determine when to stop the procedure was evaluated. We found an exponential wash out of protein from the lungs and suggest to lavage until the effluent has an OD at 405 nm of 0.04 or less, as this ensures that protein concentrations present in the normal lung are achieved.

## Patients and methods

A total of 45 lavages from patients with alveolar proteinosis were prospectively collected to study the wash out of surfactant material from the lungs during therapeutic lung lavage.

### Pediatric patients with pulmonary alveolar proteinosis

PAP was diagnosed by the characteristic histologic pattern of alveolar filling with periodic acidic Schiff positive material in open lung biopsy in all children (patients J01, J02, J03, J04). In addition the effluent from the lavages was milky and showed the characteristic cytological pattern. Patient J01 was described previously to have a heterozygous SP-C mutation[9,15]. In the other children no SP-B or SP-C mutations were detected. GMCSF autoantibodies were negative in all these patients in serum and lavage. Further clinical details of the subjects are given in Tab. 1.

**Table 1:** Patient characteristics and overview on lavages performed

Patients	Sex	Body weight	age at diagnosis	age at follow up	number of lavage sessions		number of 500 ml portions per lavage		total lavage volume recovered per lung	volume/b.w
										
		(kg)	(y)	(y)	left	right	left lung	right lung	(ml)	(ml/kg)
PAP ped. (J01)	m	8.5	1.75	5.8	9	11	9 (6.5/10)	6 (4/7)	3258 (2780/4080)	383.3
PAP ped. (J02)	f	14.5	1.75	7.8	6	6	7.5 (5.5/10)	7 (5.5/9.5)	3353 (2862/3913)	231.2
PAP ped. (J03)	f	4.5	0.33	died at age 1/3	1	1	2	8	270.3	60.1
PAP ped. (J04)	m	4.3	0.08	died at age 1/4	1	1	2	4	1018.3	236.8
										
median (25/75 percentile)		6.5 (4.4/11.5)^++^	1.0 (0.2/2.3)^++^						2007 (644.3/3199)	234 (145.7/310)
										
PAP adult (A01)	f	70.0	39.5	alive	1	1	28	29	14102	201.5
PAP adult (A02)	f	69.6	39	alive	1	0	13	0	6608	94.4
PAP adult (A03)	f	69.7	49	alive	0	1	0	35	17659	252.3
PAP adult (A04)	f	70.2	37	alive	0	1	0	27	27000	385.7
PAP adult (A05)^#^	m	80.5	51.5	alive	1	1	20	24	22000	275
PAP adult (A06)^#^	m	80.1	43.5	alive	1	1	31	39	35000	437.5
										
median (25/75 percentile)		70.1 (69.7/80.3)	42 (38/50)						19830 (10355/31000)^$$^	263.7 (248/411.6)^$^
										
Cholesterol – Pneumonitis (L01)	m	13	6.5	13.5. LTX	1	1	n.a.	2	577.5	44.4
										
control (C01)	f	13	1.5	n.a.	0	1	n.a.	n.a.	12	0.9
control (C02)	m	20	5	n.a.	0	1	n.a.	n.a.	48	2.4
control (C03)	m	16	4	n.a.	0	1	n.a.	n.a.	23	1.4
control (C04)	m	11	3	n.a.	0	1	n.a.	n.a.	17	1.6
control (C05)	f	11	2.5	n.a.	0	1	n.a.	n.a.	21	1.9
control (C06)	m	8.5	1.5	n.a.	1	0	n.a.	n.a.	21	2.5
control (C07)	f	7	0.5	n.a.	1	0	n.a.	n.a.	12.5	1.8
control (C08)	m	10	1.5	n.a.	1	0	n.a.	n.a.	14	1.4
control (C09)	m	11	2	n.a.	1	0	n.a.	n.a.	17	1.6
control (C10)	m	36	10.5	n.a.	1	0	n.a.	n.a.	65	1.8
										
median (25/75 percentile)		11 (9.3/18)	2.3 (1.5/4.5)						19 (13.3/35)	1.7 (1.4/2.2)

n.a. = not applicable. b.w. = body weight. Only diagnostic bronchoalveolar lavage. i.e. 4 ml/kg in 4 fractions; LTX = lung transplantation; data are presented as median (25/75 percentile); y = years; # = every portion consists of 1000 ml, total protein of 500 ml portions calculated; BAL = bronchoalveolar lavage; f = female; m = male; All three groups (PAP ped, PAP adult and controls) were compared by Friedmann (ANOVA), followed by Dunn's post-hoc-test: ^+^: p < 0.05, ^++^: p < 0.01, ^+++^: p < 0.001 indicate differences between pediatric PAP and adult PAP. ^§^: p < 0.05, ^§§^: p < 0.01, ^§§§^: p < 0.001 indicate differences between pediatric PAP and controls. ^$^:p < 0.05, ^$$^: p < 0.01, ^$$$^: p < 0.001 indicate differences between adult PAP and controls.

### Adult patients with pulmonary alveolar proteinosis

PAP was diagnosed by open lung biopsy (patients A01, A02, A03) or by a combination of typical clinical and radiological findings on HRCT and a diagnostic BAL showing milky fluid and abundant extracellular periodic acidic Schiff positive material on cytopreps (Patients A04, A05, A06) [16]. Clinical details of the patients are given in Tab. 1. All 6 adults patients had idiopathic PAP with high titres of GMCSF autoantibodies.

### A child with cholesterol pneumonitis and suspected alveolar proteinosis (labeled as CHOL)

The diagnosis of idiopathic cholesterol pneumonitis, associated with pulmonary alveolar proteinosis was made by open lung biopsy and the child was referred to our centre for therapeutic lavage. He had progressive respiratory distress and was oxygen dependent at that time. Two therapeutic lavages were done, one on each side. However the material obtained was not milky and thus the lavage procedure was terminated early, when almost clear fluid was recovered.

### Control children

Lavages from ten healthy children who participated in a study on the biophysical activity of surfactant [17] were used in this study as a comparison group. The children had no history of chronic respiratory symptoms or recent upper or lower respiratory tract infection. Their clinical details are given in table 1. All children were undergoing elective surgery for non pulmonary illnesses. Bronchoalveolar lavages (BAL) were performed during general anaesthesia and tracheal intubation with an endhole catheter wedged in the right lower lobe and the lavage was performed as described below. The original study of these children by BAL had been approved by the ethics committee (Nr. 97079) and written informed consent was given [17]. For the present study those lavages were used to determine the protein levels. The analysis of the therapeutic lavages was done retrospectively on samples stored after informed consent. The ethics committee had approved the anonymous usage of these samples for further variables of the surfactant system.

### Bronchoalveolar lavages and processing of the lavage fluid

Initially, in each patient, a diagnostic bronchoalveolar lavage was done. This was done either through the endhole catheter in the control children, through a bronchoscope wedged in the adult PAP patients or in the pediatric patients through a pulmonary artery catheter (Balloon Wedge Pressure Catheter, 60 cm, inner diameter 6 French = 2 mm, Arrow Inc., Reading, USA) in wedge-position on the right or left side. Normal saline (0.9% NaCl) warmed to body temperature (4 × 1 ml/kg body weight) was instilled in aliquots of 1 ml/kg bw, in adults 160 ml (8 times 20 ml) were instilled and recovered with a 20 ml syringe under manual control. The first aliquot of recovered fluid was treated separately and 2–4 ml was used for microbiological investigations. All consecutive aliquots were pooled and labeled "BAL" throughout this paper.

The therapeutic lavages in the children were done with up to 20 ml/kg b.w. aliquots of normal saline. In the small infants where it was not possible to position a double lumen endotracheal tube, a pulmonary artery catheter was introduced through an endotracheal tube and wedged in the main stem bronchus. The tightness of the fit of the balloon was continuously monitored throughout the procedure via a 1.8 or 2.3 mm flexible endoscope advanced outside the tube and positioned proximal to the balloon of the catheter. The fluid recovered was collected in consecutive 500 ml portions. In the adults, the therapeutic lavages were done similarly through one port of a double lumen endotracheal tube with 500–1000 ml aliquots of normal saline, whereas the other port was used to ventilate the contra lateral lung. The returned fluid was collected in consecutive 1000 ml aliquots.

### Analysis of proteins

Total protein concentration was measured by the method of Bradford [18]. The abundance of an abberant proform of SP-C, present in the lavages of subject J01 was determined by one dimensional SDS polyacrylamide gel electrophoresis and western blotting[9,19].

For a rapid semi-quantitative assessment of the lavage protein content, absorption measurements were performed on the native lavage samples at a wave length of 405 nm. Spectra were obtained in a spectrophotometer for wavelengths from 200 nm to 800 nm (Ultrospec 1000, Amersham Pharmacia Biotech, Uppsala, Schweden).

### Statistical analysis

Individual data points and where appropriate medians with interquartile range and range are given. Two groups were compared by Mann-Whitney test and several groups by Kruskal Wallis Anova followed by Dunn post hoc test for non-parametric variables. A p < 0.05 was considered significant. Statistical analysis was performed with Prism 4.0 (Graph Pad Software, San Diego, USA).

## Results

Therapeutic lavages were done in 4 children with median age of 1 year at diagnosis of PAP, in 6 adults (median age 42 years) and in a 6.5 year old child with cholesterol pneumonitis.

The recovered half lung lavage volume in adults was on average about 20 l per lung and in infants 2 l per lung. However, corrected for body weight, the same volume of about 250 ml/kg b.w. was used for both groups (Tab. 1). Recovery of instilled fluid in all therapeutic lavage procedures was 100 ± 10 %.

**Figure 1:**
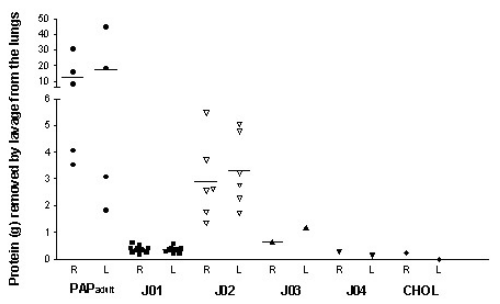
Amount of protein removed from the lungs of patients with pulmonary alveolar proteinosis of the adult (6 subjects, PAP_adult_), 4 children with PAP (J01 to J04), a child with idiopathic cholesterol pneumonitis, associated histologically with PAP (CHOL) and in 10 control children (CON). In the latter only regular diagnostic bronchoalveolar lavages were done. Each symbol represents the amount of protein recovered from a single lung lavage. L and R donates left and right sides. PAP_adult _represents total of 9 half lung lavages from patients A01 to A06. Horizontal bars indicate medians.

The amount of protein removed from the lungs by the therapeutic lavages varied substantially between subjects, but not so much within a certain subject (J01 and J02 in Fig. 1 and Tab. 2). For adult patients the removed amount of protein varied between 2 – 20 g, while the removed amount for pediatric patients was between < 0.5 to 6 g. There were no significant differences between the right and left lung (Fig. 1).

**Table 2:** Protein recovered from the lungs

Patients	concentration of protein (μg/ml)		concentration of protein (μg/ml and kg body weight)		amount of protein (mg)		amount of protein (mg/kg body weight)	
	BAL	half lung lavages	BAL	half lung lavages	BAL	half lung lavages	BAL	half lung lavages
PAP ped. (J01)	233 (195/285.1)	131 (107.5/163)	27.4	15.4 (12.6/19.2)	7.7 (7/10)	370 (287/405)	0.9	44 (34/48)
PAP ped. (J02)	306 (206.5/1149)	1010 (664/1199)	21.1	69.7 (45.8/82.7)	57 (39/134)	3214 (2262/4826)	3.9	222 (156/333)
PAP ped. (J03)	1034	2975	229.8	661	20.2	922	4.4	205
PAP ped. (J04)	352	269	81.9	62.6	5.1	223	1.2	52
								
median (25/75 percentile)	307 (207/1149)	236 (130/1010)	220 (147/252)^§^	29.7 (16.4/127)	11.7 (8/42)	545.8 (347/2618)	3 (1/4)	68.7 (43.6/329.3)
								
PAP adult (A01)	1490	924	21.3	13.2	226.7	12828	3.24	186
PAP adult (A02)	322	274	4.60	3.9	54.1	1766	0.77	26
PAP adult (A03)	135	199	1.92	2.8	21.4	3488	0.31	50
PAP adult (A04)	no data	569	no data	8.1	no data	15374	no data	220
PAP adult (A05)^#^	no data	161.5	no data	2	no data	3563	no data	45
PAP adult (A06)^#^	no data	1090	no data	13.6	no data	18820	no data	235
								
median (25/75 percentile)	846 (228/1491)	422 (180/1007)	4.6	5.8 (2.5/13.7)*	130 (38/227)^$^	5650 (1034/16850)^ns^	0.8	77 (14.1/229.8)^ns^
								
Cholesterol-Pneumonitis (L01)	136.2	157	10.5	16.8	1.7	124	0.13	9.5
								
control (C01)	47	n.a.	3.58	n.a.	0.6	n.a.	0.05	n.a.
control (C02)	58	n.a.	2.88	n.a.	2.7	n.a.	0.14	n.a.
control (C03)	77	n.a.	4.82	n.a.	1.7	n.a.	0.11	n.a.
control (C04)	82	n.a.	7.41	n.a.	1.4	n.a.	0.13	n.a.
control (C05)	47	n.a.	4.30	n.a.	1.0	n.a.	0.09	n.a.
control (C06)	85	n.a.	9.96	n.a.	1.8	n.a.	0.21	n.a.
control (C07)	97	n.a.	13.90	n.a.	1.2	n.a.	0.17	n.a.
control (C08)	77	n.a.	7.71	n.a.	1.1	n.a.	0.11	n.a.
control (C09)	65	n.a.	5.92	n.a.	1.1	n.a.	0.10	n.a.
control (C10)	49	n.a.	1.36	n.a.	3.2	n.a.	0.09	n.a.
								
median (25/75 percentile)	71 (48.2/83)		5.4 (3.2/8.8)		1.3 (1/2.3)^§§§§^		0.09	

n.a. = not applicable. only diagnostic bronchoalveolar lavage. i.e. 4 ml/kg in 4 fractions; LTX = lung transplantation; data are presented as median (25/75 percentile); y = years; # = only every second portion available. total protein calculated; BAL = bronchoalveolar lavage; f = female; m = male. Two groups were compared by Mann-Whitney-test: ns: not significant,*: p < 0.05, **: p < 0.01, ***: p < 0.001 indicate differences between pediatric and adult PAP All three groups (PAP ped, PAP adult and controls) were compared by Friedmann (ANOVA), followed by Dunn's post-hoc-test: ^+^: p < 0.05, ^++^: p < 0.01, ^+++^: p < 0.001 indicate differences between pediatric PAP and adult PAP. ^§^: p < 0.05, ^§§^:p < 0.01, ^§§§^: p < 0.001 indicate differences between pediatric PAP and controls. ^$^: p < 0.05, ^$$^: p < 0.01, ^$$$^: p < 0.001 indicate differences between adult PAP and control

The average concentration of protein in the consecutive portions of the half lung lavages was the same in adult, pediatric patients and the patient with cholesterol pneumonitis. When expressed per kg – body weight, pediatric patients had elevated concentrations (Tab. 2).

In the BAL, i.e. the diagnostic lavage, as defined in Methods, the concentrations of protein in adult and pediatric patients were clearly elevated, compared to normal children (Tab. 2). Corrected for kg – body weight, only the pediatric patients had higher levels than the controls. This difference was only about 3 – fold, too small to be reliable for diagnostic purposes.

**Figure 2:**
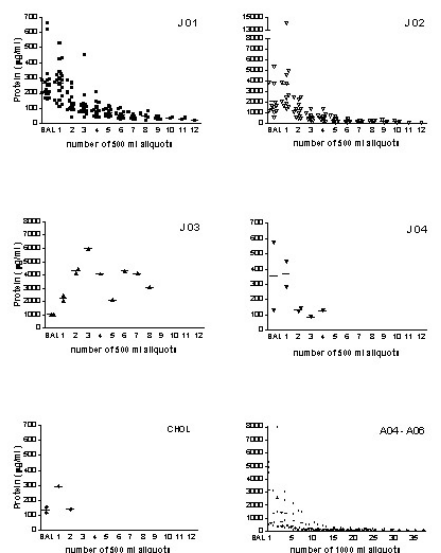
Protein concentrations in the diagnostic BAL and the consecutive 500 ml portions of lung lavages from patients with juvenile PAP (J01 – J10), a patient with cholesterol pneumonitis and PAP (CHOL) and 3 adult patients with idiopathic PAP (A04 – A06). Each symbol represents the protein concentration of one 500 ml portion lavage fluid recovered from one side. The numbers of BAL done on each side are indicated in Table 1. Horizontal bars indicate medians. Note the different scales of the protein axis.

The kinetics of the wash out followed an exponential decay function for all adult patients and for J01, J02, and J04 (Fig. 2). In patient J03, due to an insufficient procedure, because of instability of the patient, there was no real wash out function visible. This patient had in addition a severe pulmonary infection, that led together with the PAP to respiratory insufficiency and death within 8 weeks. The lavage in the child with the cholesterol pneumonitits was stopped at 1 liter due to very poor recovery of proteinous material (Fig. 2), i.e. an almost clear effluent, suggesting that the histologically suggested alveolar proteinosis was not of significant extent.

**Figure 3:**
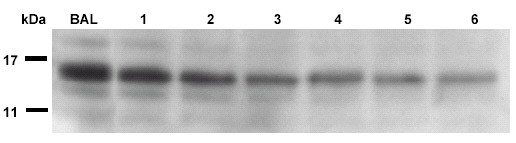
Western blot of 1 dimensional gel electrophoresis of BAL and 6 subsequent 500 ml portions of one lavage in patient J01. This patient was known to express an abberant pro-SP-C peptide in his lung. The blot was incubated with NPROSP-C Met^10^-Gln^23 ^as first and goat anti rabbit as secondary antibody to show 3 specific pro SP-C bands.

Using Western blot, clearly a wash out of an aberrant protein, i.e. pro SP-C, present in a child with PAP and SP-C mutation[15], was demonstrated. As a constant amount of protein was added to the gel, a continuous decrease of this aberrant protein, with ongoing washout, which affected all 3 aberrant pro SP-C bands equally, was observed (Fig. 3).

**Figure 4:**
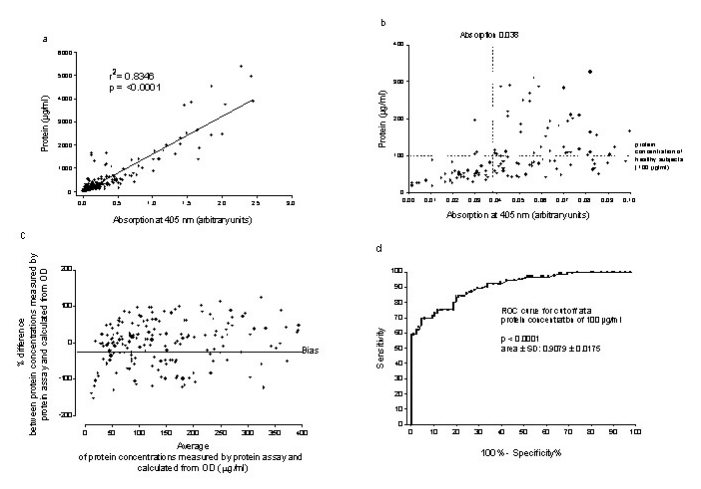
Semiquantitative estimation of the lavage protein content by measurement of its absorption at 405 nm for each 500 ml or 1000 ml portion lavage fluid fro all PAP patients (J01 – J04, A01 – A06) and the patient with cholesterol pneumonitis. a: Relationship of protein concentration and absorption at 405 nm of lung lavage fluid. There was a significant correlation between protein concentration assessed in the lavage with the Bradford assay and the absorption directly measured in the photometer. b: Zoom in on the relationship of protein concentration and absorption at 405 nm of lung lavage fluid. The maximum value of protein concentrations observed in the healthy comparison children is indicated by a dotted horizontal line. With an OD value of less than 0.038, more than 90% of the subjects with PAP had a protein concentration in their lung effluent, that was below of the healthy subjects, i.e. a protein concentration of 100 μg/ml or less. c: Bland-Altman Plot for comparison of the two methods, i.e. direct measurement of the OD of the lavage aliquot and the corresponding protein concentration, assessed by the protein assay. d: Receiver Operator Curve analysis of a cut off of of a protein concentration of 100 μg/ml. The area under the curve quantifies the overall ability of the test to discriminate those individuals with the disease, i.e. increased lavage protein concentration, and those without the disease. An area of larger than 90% (here 91%) indicates an accurate test.

An immediate estimate of the overall protein concentration would be very helpful for bed side monitoring of the lavage procedure. There was a reasonable correlation between direct OD readings, used to estimate the protein concentration from a previously made calibration curve and the precise protein concentration, as assessed by a colorimetric protein assay (Fig. 4a,b). There was consistent agreement within thumb nail error (± 100 %) (Fig. 4c). Receiver operator curves calculated for different cut-offs to stop the lavage procedure, showed a 100% specificity (i.e. the fraction correctly defined as negative) with a sensitivity (i.e. the fraction correctly defined as positive) of at least 60% at the protein concentration found in healthy subjects, i.e. 100 μg/ml or equivalent to an OD of 0.038 or less (Fig. 4b and 4d ).

## Discussion

In this study we provide detailed data on the concentrations, amounts and the wash out kinetics of proteins during therapeutic half lung lavages in infants and adults with PAP. A method was presented to easily monitor the wash out process during lavages and to determine when a physiological protein concentration is reached and a therapeutic lavage procedure may be stopped.

Since their introduction by Ramirez[20], Wasserman [21] and Seard [22], therapeutic lung lavages are the treatment of choice in patients with PAP[3,14,23,24]. While this procedures is well established and relatively easily performed in adults, therapeutic lavages in children are technically much more challenging. There are 4 reports in children[10,25-27], 7 in infants [5,28-33] and some in neonates[26,30,34]. Therefore it is not yet clear if therapeutic lung lavages are effective in treating infants with PAP. In addition there are almost no data on the protein washed out in children. Here we present the first data on such kinetics and on the amount of protein removed by whole lung lavage in small children.

In adult patients about 80 – fold higher amounts of total protein were recovered in comparison to normal whole lung lavage values which were estimated by calculation from rat lung washings[12]. Between 4 and 27.7 g were obtained, values that were similar to the 1.8 to 22 g, we found in this study. The control subjects in those studies, i.e. patients with interstitial pneumonia or alveolar cell carcinoma, had 2.8–3.4 g of protein recovered, which was about 10-fold elevated compared to rat lung washings[12]. The amount of protein removed from children with PAP was in the order of 0.4 g to 2.6 g (range 0.16 g to 5.5 g). However, when expressed per kg body weight, the same amount of protein was removed from the lungs of children and adults.

A central problem in all studies on whole lung lavages is the comparison group, as it is not appropriate to lavage normal subjects or other patients without therapeutic need. To circumvent this problem and to still be able to compare controls and PAP lavages directly, we used the regular diagnostic bronchoalveolar lavage (BAL) which was done in all subjects, before the therapeutic lavages were started, for comparison.

In our study we found protein concentrations in the diagnostic BAL that were increased 3 – fold in pediatric PAP patients and 10 – fold in adult PAP patients in comparison to controls. Despite the significant difference to control values, the result is of limited use for diagnosing PAP. There is substantial overlap with other lung diseases, like pulmonary fibrosis [35], pneumonitis[36] and bronchial asthma[37], where total protein may be elevated 2 – 5 fold, thus not allowing a clear diagnostic estimation. The protein concentrations of therapeutic lavages performed by others were 17 – 100 fold increased compared to patients with chronic bronchitis, asthma and a patient with interstitial fibrosis[38].

Until now, the protein wash out characteristic of the wash out process of a therapeutic lavage has been reported for only one patient[12]. For this reason the kinetics of the wash out is of interest. Here, for adult subjects, we show an exponential decay of protein during the procedures. For children comparable results were obtained, however at different levels of protein concentration (compare fig. 2, J01 and J02). The volumes used are about 1/10 of the ones used in adults, but when corrected for kg body weight, they were the same. A reasonable correlation between the protein concentration determined by the Bradford assay and the optical density of the lavage fluid was demonstrated. Thus, the method to monitor the estimated protein concentration in BAL fluids during lavage was evaluated further. When an OD of 0.04 or less was used as the cut off to stop the lavage procedure, the protein level was very likely to be less or in the range of the maximum protein concentration observed in healthy subjects.

Information on the progress of the wash out process from simple online and bedside monitoring may be very helpful, as can also be demonstrated in the patient with cholesterol pneumonitis. This subject had evidence from histological pattern for both cholesterol pneumonitis and PAP. The therapeutic lavage was stopped rather soon, as the effluent appeared relatively clear by visual inspection. However this may have been too early, because the protein concentration of the lavages determined after the procedure was finished, were always above 100 μg/ml. It has previously been reported in an adult patient with endogenous lipoid pneumonia due to Niemann-Pick Type B, that whole lung lavage may be successful with other diagnoses than PAP [39].

Of interest was that aberrant pro SP-C protein not normally present in lavage and found in one patient with a SP-C mutation [9], steadily decreased during the ongoing lavage, suggesting that this particular protein had accumulated over time and was efficiently removed from the alveolar space without significant replacement during the wash out.

In summary, there are considerable differences in the amount of protein washed out by whole lung lavages in children and adults with various forms of PAP. The progress of therapeutic lavage procedures and the kinetics of protein removed from the lungs during the lavage process may be continuously estimated by simple OD measurement of the effluent. This may help to make the lavage procedure more efficient, especially in young children and thus help to further optimize the technique in an age group where the procedure is technically very demanding.
